# Resection and Reconstructive Options in the Management of Dermatofibrosarcoma Protuberans of the Head and Neck

**DOI:** 10.7759/cureus.9423

**Published:** 2020-07-27

**Authors:** Aqsa Akhtar, Adeela Hussain Khan, Mamoon Rashid, Farhan Eitezaz, Haroon Ur Rashid

**Affiliations:** 1 Plastic Surgery, Shifa International Hospital, Islamabad, PAK; 2 Plastic Surgery, Queen Elizabeth Hospital, Birmingham, GBR

**Keywords:** dfsp, wide local excision, sarcoma, free flaps, head and neck, reconstruction, shifa international hospital

## Abstract

Objective

To discuss resection and various reconstructive options in patients with dermatofibrosarcoma protuberans (DFSP).

Methods

This study was conducted at Shifa International Hospital, Islamabad, Pakistan, from May 2018 to December 2019. All patients aged 20 years or above of either gender who were diagnosed to have DFSP over this period were included in the study. All the patients underwent wide local excision of the tumor under general anesthesia. A peroperative frozen section was conducted in all the cases to confirm complete excision. Immediate reconstruction was performed following the tumor excision. The choice of reconstruction, i.e. free, regional, or local flap was based on the size of the resultant defect.

Results

The mean age of the patients was 37.11 ±10.91 years. There were 12 (66.7%) males and six (33.3%) females. The mean duration of the disease was 11.22 ±2.94 months. The affected anatomical site showed that the face was involved in the majority, nine (50%) patients, followed by the scalp in four (22.2%), nape of the neck in three (16.7%), and supraclavicular region in two (11.1%) patients. In most of the cases, the free flap was observed, i.e. (n=9, 50%), followed by a regional flap in seven (38.9%), and the local flap in two (10.1%) patients.

Conclusion

Wide local excision of the disease, confirmed on frozen section, offers improved survival. Among DFSP of the head and neck, the face was found to be the affected anatomical site in half the cases. Also, reconstruction following tumor excision with a free flap is the most favorable option among patients with DFSP.

## Introduction

Dermatofibrosarcoma protuberans (DFSP) is an uncommon tumor of the skin that grows slowly [1]. The cell of origin in DFSP is a dermal stem cell or an undifferentiated mesenchymal cell with fibroblastic, muscular, and neurologic features [2].

The tumor was first described by Darier and Ferrand in 1924 [3]. It grows slowly over a period of months to years without any definitive symptoms. Hence, delay in diagnosis is not uncommon owing to the benign appearance. It infiltrates into the dermis and subcutaneous tissue but is rarely fixed to the underlying structures [4]. The tumor originates within the dermal layer of the skin and gradually extends into the local tissue to involve the subcutaneous tissue and beyond. In long-standing cases, it invades the underlying fascia, muscle, and even bone [5-6]. The local recurrence rate for patients with DFSP who undergo wide local excision (WLE) of the trunk sites ranges up to 21% [7-8]. However, in cases of head and neck, high recurrence rates have been reported, ranging from 50%-75% [8-9]. Radiation therapy has also been found to be successful in the treatment of patients with DFSP recently [10].

Owing to its characteristic fibroblastic cellular features, DFSP is readily identifiable on frozen-section examination with hematoxylin and eosin (H&E) staining. These features include a cigar-shaped nuclear outline, a cartwheel or storiform pattern of nuclei arranged in irregular strands and whorls, and fibrotic stroma [1].

These cases present a challenge for the soft tissue coverage following an oncologically safe resection. The aim of this study is to discuss the resection and various reconstructive options for DFSP of the head and neck.

## Materials and methods

This prospective observational study was conducted at Shifa International Hospital, Islamabad, from May 2018 to December 2019.

Ethical approval was obtained from the institutional review board (IRB #1129-405-2018) of the hospital prior to conducting the study. Moreover, signed informed consent was also obtained from all the patients after explaining the pros and cons of the study.

All patients who had biopsy-proven DFSP in the head and neck region over this period were included in the study.

The head and neck region was described: anteriorly - above clavicles; posteriorly - up to the first thoracic spine; and laterally - above the shoulder.

Pre-operative evaluations

Most of the patients were referred to our hospital from primary clinics after an established diagnosis of definitive surgical excision. 

Pre-operative workup included the biochemical laboratory evaluation and an MRI to know the extent of the lesion.

Operative technique

All patients underwent wide local excision of the tumor under general anesthesia. The resection margins were 2-3 cm and were tailored according to the involvement of the critical area. Peroperative frozen section was conducted in all the cases to confirm complete excision with negative margins. Immediate reconstruction was performed following the tumor excision.

The choice of reconstruction, i.e. free, local, or regional flap was based on the size of the defect and the area involved.

This information, along with demographic characteristics like age, gender, duration of disease, size of defects, margins, and anatomical site, was noted. 

Statistical analysis was performed using Statistical Package for the Social Sciences (SPSS) version 24 (IBM Corp., Armonk, NY). Mean and standard deviation was calculated for quantitative variables like age, duration of disease, margins, and size of defects. Frequency and percentages were calculated for qualitative variables like gender, anatomical site, and reconstruction.

## Results

The mean age of the patients was 37.11 ±10.91 years. There were 12 (66.7%) males and six (33.3%) females. The mean duration of the disease was 11.22 ±2.94 months. The affected anatomical site showed that the face was involved in the majority (9 (50%)) patients, followed by the scalp in four (22.2%), the nape of the neck in three (16.7%), and the supraclavicular region in two (11.1%) patients. The frequency of reconstruction showed that the free flap was found in the majority (nine (50%)), followed by the regional flap in seven (38.9%), and the local flap in two (10.1%) patients. The mean margin was 2.55 ±0.51. (Table 1). The detailed demographic and clinical data of dermatofibrosarcoma protuberans cases of head and neck is showed in Table 2. Of nine patients with a free flap, the radial forearm free flap was observed in five (55.5%), the anterolateral thigh flap in four (44.4%) patients. Of seven patients with a regional flap, the myocutaneous trapezius flap was observed in five (71.42%), and the myocutaneous latissimus dorsi flap in two (28.57%) patients. Whereas, of the two cases with a local flap, both were of the scalp rotation flap. (Table 2).

**Table 1:** Baseline characteristics of the patients (n=18)

	mean ±SD	
Age, years	37.11 ±10.91	
Duration of disease, months	11.22 ±2.94	
Margins	2.55 ±0.51	
	n	%
Gender		
Male	12	66.7
Female	6	33.3
Anatomical Site		
Face	9	50
Scalp	4	22.2
Nape of the Neck	3	16.7
Supraclavicular Region	2	11.1
Reconstruction		
Free Flap	9	50
Regional Flap	7	38.9
Local Flap	2	10.1

**Table 2:** Detailed demographic and clinical data of dermatofibrosarcoma protuberans cases of the head and neck

Case	Age, years	Gender	Duration of Disease, months	Anatomical Site	Region	Reconstruction	Size of defects	Margins
1	48	Male	6	Posterior scalp (Occipital)	Scalp	Regional flap (Pedicled myocutaneous trapezius flap)	11 x 5	3
2	55	Male	11	Parieto-occipital region	Scalp	Local scalp (Scalp rotation flap)	8 x 6	3
3	48	Female	8	Right cheek	Face	Distant flap (Radial forearm free flap)	15 x 8	2
4	28	Female	17	Forehead	Face	Distant flap (Radial forearm free flap)	9 x4	2
5	32	Male	12	Nape of the neck	Nape of the Neck	Regional flap (Pedicled myocutaneous trapezius flap)	10 x 5	3
6	48	Male	10	Left temporal region	Face	Distant flap (Anterolateral thigh free flap)	15 x 8	3
7	22	Female	9	Left supraclavicular region	Supraclavicular Region	Regional flap (Pedicled myocutaneous latissimus dorsi flap)	11 x 7	3
8	28	Male	14	Forehead	Face	Distant flap (Radial forearm free flap)	15 x 1	3
9	47	Male	7	Left cheek	Face	Distant flap (Radial forearm free flap)	7 x 4	2
10	52	Male	9	Right temporal region	Face	Distant flap (Anterolateral thigh free flap)	12 x 6	2
11	32	Male	13	Nape of the neck	Nape of the Neck	Regional flap (Pedicled myocutaneous trapezius flap)	9 x 7	3
12	21	Female	12	Left supraclavicular region	Supraclavicular Region	Regional flap (Pedicled myocutaneous latissimus dorsi flap)	9 x 7	2
13	45	Male	12	Right temporal region	Face	Distant flap (Anterolateral thigh free flap)	12 x 8	2
14	35	Male	14	Nape of the neck	Nape of the Neck	Regional Flap (Pedicled myocutaneous trapezius flap)	10 x 4	3
15	40	Male	16	Posterior scalp (Occipital)	Scalp	Regional flap (Pedicled myocutaneous trapezius flap)	12 x 4	3
16	24	Female	10	Left cheek	Face	Distant flap (Anterolateral thigh free flap)	12 x 6	2
17	29	Male	12	Left cheek	Face	Distant flap (Radial forearm free flap)	11 x 8	2
18	34	Female	10	Parieto-occipital region	Scalp	Local scalp (Scalp rotation flap)	9 x 6	3

As far as the duration of the disease is concerned, the face and nape of the neck were the most affected anatomical site in most patients with >11 months of duration of disease.

## Discussion

Dermatofibrosarcoma protuberans is a rare, intermediate-grade, soft tissue sarcoma having characteristically progressive local growth and a propensity for extensive subclinical involvement with local recurrence. WLE of the tumor with histologically negative margins is the fundament of treatment [11].

In the current study, all patients were treated with wide local excision. It is reported that WLE produces near or equivalent postoperative defect sizes and oncological safety, suggesting less morbidity decreasing the chances of recurrence [11-12]. In large and extensive tumors, Mohs micrographic surgery (MMS) can be technically challenging, despite its potential advantage of sparing healthier tissue. Excising large tumors under local or tumescent anesthesia, both typically used in MMS, can be difficult. For these reasons, WLE alone or in combination with MMS in a multidisciplinary approach may be necessary, and it has been proven effective by DuBay and colleagues [11].

In our study of DFSP of the head and neck, the affected anatomical site showed that face was involved in majority 50% patients, followed by the scalp in 22.2%, the nape of the neck in 16.7%, and the supraclavicular region in 11.1% patients. Findings from most studies report that the trunk was involved in the majority of the cases (31.7%), followed by the head in 28.9%, the lower limb in 18.3%, and the upper limb in 16.9% patients [13]. Similarly, Veronese et al. also reported the trunk as the affected anatomical site in the majority of the cases, followed by the back, legs, arms, and head and neck, whereas genitalia was reported as the affected anatomical site in 1one patient [14].

"Whatever is excisable, is reconstructable!" are the assured words of reconstructive surgeons today. Since the introduction of microsurgery, dramatic reconstructive options have been available for patients following tumor excision. The use of free flap offers effective and definitive repair in most cases [15].

In our study, the free flap was observed in 50%, followed by the regional flap in 38.9%, and the local flap in 10.1% of patients. On further assessment, it was noted that the use of the free flap was mostly required for defects of the face, regardless of the defect size. However, in other areas, e.g., the posterior scalp, nape of the neck, and the supraclavicular region, local or regional flaps were used for reconstruction.

The choice of the reconstructive plan should depend on the aim to replace the lost tissue and provide near-normal functional and aesthetic outcomes while maintaining a natural balance [16].

**Figure 1:**
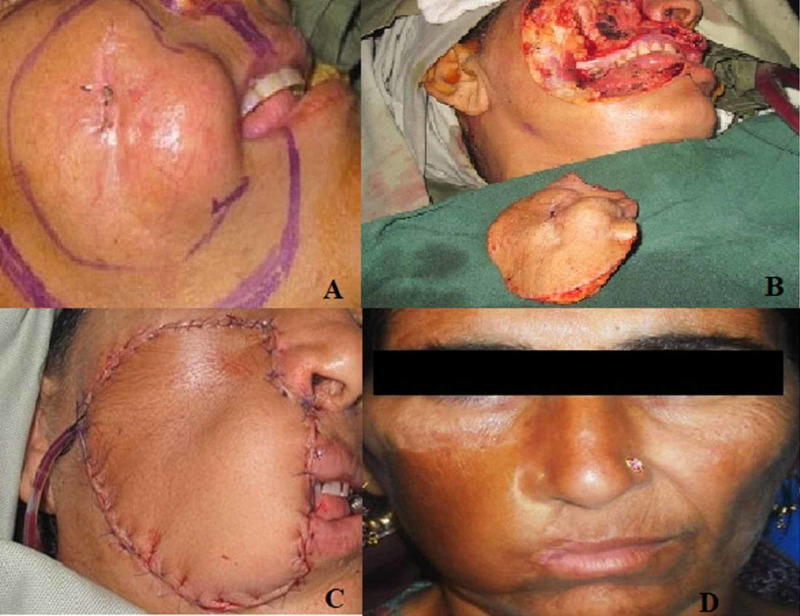
A: Long-standing DFSP of the cheek, involving the underlying buccal mucosa. B: Resection specimen with the defect. C: Reconstruction using the radial forearm free flap. D. Follow-up DFSP: dermatofibrosarcoma protuberans

Defects of the cheek were reconstructed using either a radial forearm free flap or anterolateral thigh flap, depending on the depth of the defect (Figure 1). Because of the paucity of the tissue in the face and consideration of the aesthetics, the distant free flap was used for the purpose of reconstruction in the face to achieve improved facial outlook.

**Figure 2:**
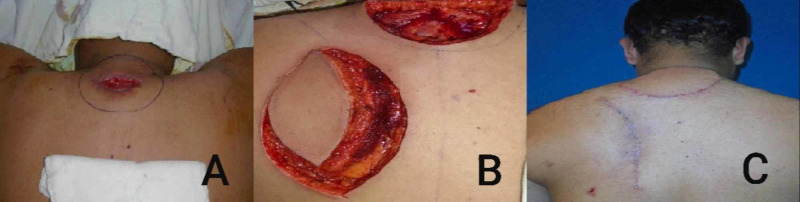
A: DFSP - nape of the neck. B: Defect and elevated trapezius myocutaneous flap. C: Follow-up DFSP: dermatofibrosarcoma protuberans

**Figure 3:**
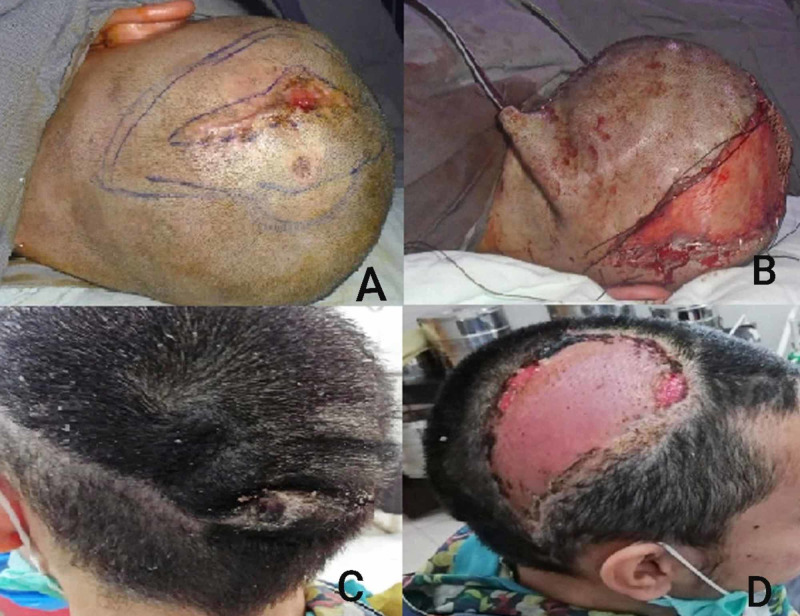
A: DFSP of scalp. B: Reconstruction with a scalp rotation flap. C&D: Follow-up DFSP: dermatofibrosarcoma protuberans

In the clavicular region and the defects over the nape of the neck, the options of the regional flap were possible (Figure 2). However, in two cases involving the parieto-occipital region of the scalp, a scalp rotation flap was used and the resultant donor area was resurfaced using a split-thickness skin graft (STSG) (Figure 3). These patients are planned for later tissue expansion and resurfacing.

None of the patients experienced free, local, or regional flap failure.

The prognosis of DFSP is generally good, but this tumor is associated with locally aggressive biology, especially of the head and neck. Although tumors in this location affected 18.3% of the study population, they were associated with 36.7% of the recurrences, likely owing to the difficulty in achieving wide margins in these anatomic locations [17-18].

Prognosis of the disease is excellent after adequate surgical clearance. Overall survival rates are 93% to 100% [12]. The findings of this study could be highlighted in light of the limitation that this study was a single-center observational study. Moreover, this study only included patients who were treated with WLE. In addition, other important characteristics like clinical variety (protruding form, morphea-like form, and congenital), local recurrence, and relapse rate were not included. Despite these limitations, this study is a significant effort in reporting ample data on DFSP of the head and neck from Pakistan. Though the sample size of the current study is not large, the prospective nature of the study has made the study significant, as most of the previous studies were conducted retrospectively or merely a case report.

Meanwhile, during the course of the study, none of the patients reported any signs recurrence clinically. The individual cases were not studied, however, because of the limited duration of the study. All the patients are intended to be followed further for any clinical or radiological evidence of recurrence for the next five years.

## Conclusions

Head and neck DFSP is not a common tumor but because of its indolent nature, it presents a local problem. The overall survival rate is excellent. A surgical resection margin of 2-3 cm from the gross tumor boundary, further confirmed on frozen section, has a favorable impact on survival. Considering the critical location of the disease on the head and neck and the paucity of the surrounding tissue, the free flap is the preferable reconstructive choice to restore the functional and cosmetic outlook.
